# Single versus dual hypothermic oxygenated perfusion in liver transplantation: a call for risk-matched outcome analyses

**DOI:** 10.1097/JS9.0000000000002376

**Published:** 2025-04-09

**Authors:** Omer F. Karakaya, Sangeeta Satish, Philip C Müller, Philipp Dutkowski, Andrea Schlegel

**Affiliations:** a Department of Inflammation & Immunity, Lerner Research Institute, Cleveland Clinic, Cleveland, Ohio, USA; b Transplantation Center, Cleveland Clinicss, Cleveland, Ohio, USA; c Department of Surgery, Clarunis – University Digestive Health Care Centre Basel, Basel, Switzerland

**Keywords:** biliary complications, hepatic artery, hypothermic oxygenated perfusion, ischemia-reperfusion injury, liver transplantation, portal vein

Dear Editor,

We commend Pereyra *et al*^[^1^]^ for their recent study comparing static cold storage (SCS) with single (sHOPE) and dual hypothermic oxygenated perfusion (dHOPE) in liver transplantation. This single-center, retrospective analysis from Austria evaluated post-transplant biliary complications primarily in donation after brain death (DBD) livers, with 69 in the SCS group, 76 in sHOPE (through portal vein only), and 102 in the dHOPE cohort (through portal vein and hepatic artery). The study found that HOPE treatment generally reduced biliary complication rates compared to SCS, with dHOPE further lowering anastomotic stricture (AS) rates and required surgical interventions compared to sHOPE.

We applaud the authors for their work and would like to share some thoughts on certain methodological considerations and design choices that may influence the interpretation of the study findings.

First, as acknowledged by the authors, this retrospective study did not use predetermined donor or recipient criteria for selecting one of the two HOPE modalities. Livers were allocated to single HOPE when multiple hepatic arteries were present, or an aortic patch was lacking with the need for reconstructions to enable arterial cannulation. This lack of standardization introduces potential selection bias, which may affect the reliability of outcome comparisons between the sHOPE and dHOPE cohorts.

Second, the number of donation after circulatory death (DCD) livers in the overall cohort is low (n = 10) and unevenly distributed across study groups, with 5 in the SCS arm (7.7%), 4 in single HOPE (5.5%), and only 1 in the dual HOPE group (1%). This risk distribution does not reflect typical clinical settings in centers with active DCD programs. Next, previous studies show substantial benefits but essentially no difference between both HOPE modalities in reducing biliary complications, particularly in DCD cohorts^[^2,3^]^. Importantly, while sHOPE and dHOPE have been effective in reducing non-anastomotic stricture (NAS), a similar number of anastomotic biliary strictures were repeatedly reported^[^4,5^]^. Larger DCD samples would be needed for more robust conclusions on the comparative effectiveness of these perfusion strategies.

**HIGHLIGHTS**

- Pereyra *et al*’s retrospective study claims a reduction in biliary complications with dual compared to single hypothermic oxygenated perfusion in a cohort of mainly benchmark donation after brain death livers for transplantation.
- Limited and unequal donation after circulatory death liver distribution among study groups underscores limitations in donor risk standardization.
- Risk-matched analyses are needed to improve data robustness comparing different hypothermic oxygenated perfusion modalities.

The third and main limitation is the absence of a risk-matched analysis. Comparing outcomes across different groups without accounting for varying donor and recipient risk factors but also surgical parameters and posttransplant pressure support and other recipient confounders, can result in misleading conclusions. Risk-matched analyses would better control for the multiple confounders and allow for a more accurate assessment of the true effects of sHOPE and dHOPE. Despite the generally rather low donor risk with mainly DBD grafts in the here presented study, the single HOPE group had slightly higher Donor Risk Index (DRI) and longer cold ischemia time prior to HOPE-treatment^[^1^]^. Of note, the same Austrian group has published higher perfusate syndecan-1 values in their sHOPE compared to dHOPE cohort, supporting the different donor risk profiles^[^6^]^.

Interestingly, both HOPE arms reduced the NAS rate to 3.9% from 5.8% in the SCS group, despite a higher number of DCD grafts in the single HOPE group (4 DCD in sHOPE vs. only 1 in dHOPE). Authors also describe a higher AS rate (13.2%) with more surgical and endoscopic interventions in the sHOPE group compared to 6.9% after dHOPE^[^1^]^. While such interventions accumulate during the follow-up period, it is important to mention the much shorter median posttransplant follow-up in dHOPE with 15.1 months (IQR: 7.7–36.9) compared to 23.3 months (IQR: 11.0–34.8) after sHOPE.

Fourth, the study lacks liver risk stratification, which would clarify liver quality and might explain outcome differences between sHOPE and dHOPE groups. Numerous large cohort studies over the past decade have examined outcomes with both HOPE modalities (Table 1).

**Table 1:** Overview on current literature on effect of single and dual HOPE on biliary complications in clinical studies with liver transplantation

Type of study, center (n)	Author, year (location)	Graft type, cohort (n)	Perfusion duration (median)	Duration of follow-up	Primary outcome and main findings	Anastomotic biliary strictures	Non-anastomotic biliary strictures	Discussion/ limitations
sHOPE								
RCT, Multicenter (10)	Schlegel *et al* (Switzerland, France, Netherlands, UK, Belgium, Austria)^[^4^]^	DBD/ECD	95.5 min	12 months	Primary outcome: no statistical difference in incidence of 1 or more Clavien Dindo complications ≥ 3 with sHOPE No liver-related graft loss and retransplantation with sHOPE Less EAD compared to SCS cohort (% liver-related graft complication rate)	SCS:21.2% (n = 18/85)	SCS: 3.5% (n = 3/85)	sHOPE was associated with fewer severe liver-related complications (Clavien > IIIb) in DBD grafts. Further studies are recommended to confirm these findings.
							sHOPE: 1.2% (n = 1/85, no graft loss, conservative treatment)	No DCD grafts used
						sHOPE: 16.5% (n = 14/85)		
		SCS (85) sHOPE (85)						
RCT, single center	Ravaioli *et al* (Italy)^[^9^]^	DBD/ECD	145 min	12 months	Primary outcome: lower EAD (13% vs 35%, *P* = 0.007) with sHOPE No PNF, higher 1-year graft survival (*P* = 0.03) in sHOPE cohort Lower readmission and overall complication (*P* = 0.04 and *P* = 0.03, respectively) compared to SCS	Hepatic Biliary or Vascular complications; sHOPE: 16% (n = 9/55), SCS: 22% (n = 12/55)		No DCD livers, combined presentation of biliary and vascular complications; Biliary complications not further investigated for AS, NAS
		SCS (55) sHOPE (55)						
RCT, multicenter (4)	Czigany *et al* (Germany, Czech Republic)^[^10^]^	DBD/ECD	145 min	12 months	Primary outcome: statistically significant reduction in peak ALT levels within 7 days (*P* = 0.03) Shorter ICU and hospital stay (*P* = 0.045 and *P* = 0.002, respectively) with sHOPE Less major complications and cumulative complications (*P* = 0.036 and CCI *P* = 0.021), lower estimated costs after transplantation (*P* = 0.016) in sHOPE compared to SCS	Biliary complications (clinical; radiological): SCS: 26% (n = 6/23), sHOPE: 17% (n = 4/23)		Study was not powered for biliary complications, No DCD livers, no specific information on NAS
		SCS (23) sHOPE (23)						
Retrospective, multicenter (17)	Schlegel *et al* (Europe and North America)^a^	DCD (2,219) (Benchmark/Low-risk 1,012)	120 min	12 months	Significantly reduced non anastomotic biliary strictures (*P* = 0.0071) with sHOPE	SCS: 22.1%, sHOPE: 26.5%	SCS: 22.06%, sHOPE: 4.1% (*P* = 0.0071)	Further prospective studies are needed to confirm benchmark efficacy
		SCS (87) sHOPE (49)						
Case-control cohort study (IV)	Rayar *et al* (France)^b^	DBD/ECD	120–240 min (ideal)	12 months	Reduced peak transaminases and less EAD with sHOPE Shorter stay in hospital and in ICU	Anastomotic strictures or leaks:	SCS: 1.4% (n = 1/69; ischemic necrosis)	The perfusion cohort was selected prospectively, while the control group was selected retrospectively, and biliary complications were not the primary endpoint
		SCS (69) sHOPE (25)						
						SCS: 10.1% (n = 7/69)		
							HOPE: 0% (n = 0/25)	
						HOPE: 8% (n = 2/25)		
	Schlegel *et al* (UK, Switzerland)^[^3^]^	DBD, DCD	120 min	5 years	Less PNF, HAT and NAS Improved 5-year survival of HOPE-treated cohort (extended DCD liver grafts)	SCS DCD: 18% (n = 9/50)	SCS: 22% (n = 11/50) with 10% (n = 1/69) graft loss	Retrospective design, different implantation techniques and overall higher risk in Zurich DCD cohort
		sHOPE-DCD (50) SCS-DBD (50) control SCS-DCD (50)						
						HOPE: 24% (n = 12/50), 1 biliary leak each group (2%)		
							sHOPE: 8% (n = 4/50) with 0% graft loss	
	Ravaioli *et al* (Italy)^c^	Extended DBD HOPE (10) SCS (30)	132 min	12 months	No PNF and significantly lower rate of EAD, lower transaminases with HOPE 100% graft survival	SCS: 10% (n = 3/30)	No specific information provided	Rather low case number, matched cohort study, DBD
						HOPE: 10% (n = 1/10)		
Retrospective case-control	Dutkowski *et al* (Switzerland)^[^2^]^	DCD, DBD	129 min	HOPE-DCD: 448 days,	Increased 1-year graft survival rate (*P* = 0.035) with sHOPE Less biliary complications (*P* = 0.042) and less initial reperfusion injury	Anastomotic Strictures or leak,	DCD-SCS: 22% (n = 11/50)	Retrospectıve design
						DCD-SCS: 24% (n = 12/50)		
				SCS-DCD: 528 days,				
						HOPE: 20% (n = 5/25)		
				SCS-DBD: 1530 days				
						DBD-SCS: 20% (n = 10/50)		

		sHOPE-DCD (25) SCS-DCD (50) SCS-DBD (50)						
							sHOPE: 0%	
							DBD-SCS: 4% (n = 2/50)	
**Studies combining sHOPE and dHOPE (single and dual)**								
Case-control cohort study	Koch *et al* (Munich/ Germany)^d^	DBD (146) sHOPE (73) dHOPE (73)	sHOPE: 154.15 min	sHOPE: 872 days	dHOPE did not show a notable benefit over sHOPE in terms of patient survival (*P* = 0.990) and graft survival (*P* = 0.754) Non-significant difference between sHOPE and dHOPE cohorts in NAS (*P* = 0.574) and hospital length of stay (*P* = 0.331) for DBD liver grafts. Non-significant difference in ET-DRI (*P* = 0.957)	No specific information provided	sHOPE: 10.96% (n = 8/73)	First head-to-head comparison, includes only DBD with an advanced risk and an ET-DRI of 2.12 in both groups.
							dHOPE: 8.22% (n = 6/73)	
				dHOPE: 367 days				
			dHOPE: 178.94 min					
Case-control cohort study	Pereyra *et al* (Austria/ Vienna)^[^1^]^	DBD (237)	dHOPE: 150 min	sHOPE 23.3 months	HOPE (total): Reduced biliary complications and shorter hospital stay dHOPE: Independently associated with reduced number of surgical revision case	SCS: 14.5% (n = 10/69)	SCS: 5.8% (n = 4/69)	Not risk matched, unequal risk distribution, non-homogenous distribution of DCD livers, not accounted for many confounders
		SCS (69) sHOPE (76) dHOPE (102)						
		DCD (10)						
		SCS (5) sHOPE (4) dHOPE (1)						
		DBD-sHOPE (381)						
			sHOPE: 150 min			sHOPE: 13.2% (n = 10/76)	sHOPE: 3.9% (n = 3/76)	
				dHOPE 15.1 months				
						dHOPE: 6.9% (n = 7/102)	dHOPE: 3.9% (n = 4/102)	
Retrospective, multicenter (22), not controlled	Eden *et al* (Europe and North America)^[^7^]^	Benchmark (42) Standard (85) Extended	Total:142 min	12 months	Higher graft survival in DBD livers (*P* < 0.01) Non-significant difference between sHOPE and dHOPE cohorts in death censored graft survival (*P* = 0.73) No differences with different perfusion devices in outcomes (*P* = 0.29) Lower PNF-related (2.3%) and IC-related (0.4%) graft loss in DBD livers compared to DCD cohort (5% PNF and 4.1% NAS)	DCD (26%)	Overall incidence NAS 2.5% in DBD, 12.4% in DCD (*P* < 0.001)	Largest cohort with HOPE, no SCS control group, no matched analysis
			DBD:150 min (Benchmark: 146 min; Standard: 155 min; Extended criteria: 146 min)					
						DBD (13%, *P* < 0.001)		
		Criteria (254)						
			DCD:134 min (Low risk: 133 min; High-risk: 130 min; Futile: 142 min)					
		DBD-dHOPE (387)						
		Benchmark (57) Standard (91) Extended						
		Criteria (239)						
		DCD-sHOPE (183)						
		Low risk (40) High-risk (88) Futile (55)						
		DCD-dHOPE (115)						
		Low risk (58) High-risk (51) Futile (6)						
**DUAL/dHOPE**								
RCT, single center	Grat *et al* (Poland)^e^,^f^	DBD	120 min	90 days	Primary outcome: no significant decrease in MEAF score with dHOPE Benefits are limited to high-risk donors (DRI>1.70), significantly lower MEAF (*P* = 0.037) and CCI (*P* = 0.05)	5-year follow up:	dHOPE: 0%	Lack of power for biliary complication analysis
							SCS:11.1 (*P* = 0.1)	
						dHOPE: 19.9%		
						SCS: 33.7% (*P* = 0.2)		
		SCS (78) dHOPE (26)						
RCT, multicenter (9)	Panayotova *et al* (USA)^g^	DBD (136)	168 min	12 months	Primary outcome: Lower EAD (11.1% vs 16.4%) and L-GrAFT7 (3.4% vs 4.5%) with dHOPE Reduced biliary stricture cases compared to SCS cohort (16.4% vs 6.3%)	HMP-O_2_: 11.1% (n = 7/63)	dHOPE: 0%	Lack of blinding and potential variability in HMP-O_2_ protocol across centers
		SCS (73) HMP-O_2_ (63)					SCS: 5.5% (4/73)	
		DCD (15)						
		SCS (10) HMP-O_2_ (5)						
						SCS: 16.4% (n = 12/73)		
RCT, multicenter (6)	Van Rijn *et al* (Netherlands)^[^5^]^	SCS (78) dHOPE (78)	132 min	6 months	Primary outcome: Significant reduction in NAS (*P* = 0.03) Reduced number of required interventions	SCS 28.2% (n = 22/78)	SCS: 18% (n = 14/78)	Powered for NAS, fairly short follow up (6 months)
							dHOPE: 6% (n = 5/78)	
						dHOPE: 29.5% (n = 23/78)		
Retrospective, matched analysis	Patrono *et al* (Italy)^h^	DBD/ECD	138 min	dHOPE: 22 months	Significant reduction in postop grade >2 complications and EAF (*P* = 0,046 and *P* = 0.24, respectively) Improved patient and graft survival	dHOPE: 19%(n = 23/121),	SCS: 4.8% (n = 35/723),	Retrospective design and lack of randomization
		SCS (723) dHOPE (121)						
				SCS: 47.3 months		SCS: 13%(n = 94/723)	dHOPE: 4.1% (n = 5/121)	
Retrospective, matched analysis	Patrono *et al* (Italy)^i^	DBD/ECD macro-steatotic	186 min	6 months	Decreased rate of post-reperfusion syndrome Lower rate of acute kidney injury grade 2-3 Reduced EAD rate and lower recipient transaminases	SCS: 12% (n = 6/50)	SCS: 8% (n = 4/50), 2 symptomatic patients	DBD grafts, Selective use of HOPE
						dHOPE: 16% (n = 4/25)		
		dHOPE (25) SCS extended						
							dHOPE: 8% (n = 2/25), asymptomatic	
		DBD (matched) (50)						
Case-control cohort study	Van Rijn *et al* (Netherlands)^j^	DCD	126 min	12 months	Increased ATP in dHOPE-treated livers(restoration) Lower transaminases Protection from reperfusion injury in biliary tree Less complications	SCS: 15% (n = 3/20)	SCS: 45% (n = 9/20) with 2 biliary necroses, 5 retransplantations;	Small sample size
		dHOPE (10) SCS (20)						
						dHOPE: 20% (n = 2/10)		
							dHOPE: 10% (n = 1/10), no retransplantations	
Case-control cohort study	Guarrera *et al* (USA)^k^	ECD (DCD and DBD)	228 ± 54 min	12 months	Significantly lower biliary complications (*P* = 0.016) Lower transaminases (ALT at pod1, *P* = 0.049) Shorter hospital stays (*P* = 0.001)	Overall biliary complications: dHOPE:13% (n = 4/31)	No specific information on NAS	No analysis of Non-anastomotic strictures
		dHOPE (31) SCS (30)						
						SCS:43% (n = 13/30) *P* = 0.001.		
						Biliary stricture:		
						dHOPE: 10% (n = 3/31),		
						SCS: 33% (n = 10/30, *P* = 0.031		


a Schlegel A, van Reeven M, Croome K, Parente A, Dolcet A, Widmer J, et al. A multicentre outcome analysis to define global benchmarks for donation after circulatory death liver transplantation. J Hepatol. 1 February 2022;76(2):371–82.

b Rayar M, Beaurepaire J, Bajeux E, Hamonic S, Renard T, Locher C, et al. Hypothermic Oxygenated Perfusion Improves Extended Criteria Donor Liver Graft Function and Reduces Duration of Hospitalization Without Extra Cost: The PERPHO Study. Liver Transplantation [Internet]. 2021;27(3). Available from: https://journals.lww.com/lt/fulltext/2021/03000/hypothermic_oxygenated_perfusion_improves_extended.9.aspx

c Ravaioli M, De Pace V, Angeletti A, Comai G, Vasuri F, Baldassarre M, et al. Hypothermic Oxygenated New Machine Perfusion System in Liver and Kidney Transplantation of Extended Criteria Donors: First Italian Clinical Trial. Sci Rep. 1 December 2020;10(1).

d Koch DT, Tamai M, Schirren M, Drefs M, Jacobi S, Lange CM, et al. Mono-HOPE Versus Dual-HOPE in Liver Transplantation: A Propensity Score-Matched Evaluation of Early Graft Outcome. Transplant International [Internet]. 5 February 2025;38. Available from: https://www.frontierspartnerships.org/articles/10.3389/ti.2025.13891/full

e Grąt M, Morawski M, Zhylko A, Rykowski P, Krasnodębski M, Wyporski A, et al. Routine End-ischemic Hypothermic Oxygenated Machine Perfusion in Liver Transplantation From Donors After Brain Death: A Randomized Controlled Trial. Ann Surg [Internet]. 2023;278(5). Available from: https://journals.lww.com/annalsofsurgery/fulltext/2023/11000/routine_end_ischemic_hypothermic_oxygenated.5.aspx

f Morawski M, Zhylko A, Rykowski P, Krasnodębski M, Hołówko W, Lewandowski Z, et al. Routine end-ischemic hypothermic machine perfusion in liver transplantation from donors after brain death: results of two-year follow-up of a randomized controlled trial. International Journal of Surgery. 11 July 2024;

g Panayotova GG, Lunsford KE, Quillin RC, Rana A, Agopian VG, Lee-Riddle GS, et al. Portable hypothermic oxygenated machine perfusion for organ preservation in liver transplantation: A randomized, open-label, clinical trial. Hepatology. 1 May 2024;79(5):1033–47.

h Patrono D, Cussa D, Sciannameo V, Montanari E, Panconesi R, Berchialla P, et al. Outcome of liver transplantation with grafts from brain-dead donors treated with dual hypothermic oxygenated machine perfusion, with particular reference to elderly donors. American Journal of Transplantation. 1 May 2022;22(5):1382–95.

i Patrono D, Surra A, Catalano G, Rizza G, Berchialla P, Martini S, et al. Hypothermic Oxygenated Machine Perfusion of Liver Grafts from Brain-Dead Donors. Sci Rep. 1 December 2019;9(1).

j van Rijn R, Karimian N, Matton APM, Burlage LC, Westerkamp AC, van den Berg AP, et al. Dual hypothermic oxygenated machine perfusion in liver transplants donated after circulatory death. British Journal of Surgery. 1 June 2017;104(7):907–17.

k Guarrera J V., Henry SD, Samstein B, Reznik E, Musat C, Lukose TI, et al. Hypothermic machine preservation facilitates successful transplantation of “orphan” extended criteria donor livers. American Journal of Transplantation. 1 January 2015;15(1):161–9.

Abbreviations: ALT: alanine aminotransferase; AS: anastomotic biliary stricture; ATP: adenosine trisphosphate; CCI: comprehensive complication index; DBD: donation after brain death; DCD: donation after circulatory death; d-HOPE: dual HOPE; EAD: early allograft dysfunction; ECD: extended criteria graft; ET-DRI: Eurotransplant Donor risk index; HAT: hepatic artery thrombosis; HOPE: hypothermic oxygenated perfusion; L-Graft7: liver graft assessment following transplantation (day 7); MEAF: model for early allograft function; NAS: non anastomotic biliary strictures; PNF: primary non function; SCS: static cold storage; sHOPE: single HOPE.

Current literature, including three large randomized controlled trial (RCTs) and two cohort studies with over 237 DCD liver transplants, shows that sHOPE reduces NAS compared to SCS (Table 1). In a recent multicenter study by Eden *et al*, involving 434 DCD liver transplants stratified by UK-DCD risk score, HOPE-treated transplants had excellent long-term outcomes across all risk levels, including high-risk DCDs^[^7^]^. No significant differences were found between sHOPE and dHOPE across European centers, indicating similar protective effects for both methods when perfusate was highly oxygenated (pO_2_ >70 kPa), the key factor in protecting biliary endothelial cells from ischemia-reperfusion injury^[^8^]^. Given the venous and arterial supply to the extrahepatic biliary tree, portal vein HOPE alone appears to deliver sufficient oxygen to protect sensitive cholangiocytes.

In summary, while this study contributes to the literature on HOPE in liver transplantation, its methodological limitations suggest cautious interpretation. Future studies addressing these issues may clarify the benefits of both HOPE strategies.

## Data Availability

None.
